# Sustainable energy supply and consumption by 2050 and outlook towards the end of the century: Possible scientific breakthroughs

**DOI:** 10.1007/s13280-015-0735-8

**Published:** 2015-12-14

**Authors:** Lennart Bengtsson, Elisabeth Rachlew, Friedrich Wagner

**Affiliations:** Max Planck Institute for Meteorology, Hamburg, Germany and Environmental Systems Science Centre, Reading, UK; Department of Physics, Royal Institute of Technology, 10691 Stockholm, Sweden; IPP, Max Planck Gesellschaft, Greifswald, Germany

## Introduction

A project launched by the European Academies’ Science Advisory Council (EASAC) in 2013 identified possible areas of scientific breakthroughs in energy supply and consumption with a long-term perspective up to and beyond 2050.

The project facilitated interactions and information sharing among scientists in Europe and worldwide through electronic communications and two dedicated workshops. A steering committee with eighteen scientists from eleven countries was appointed by the EASAC participating academies (Box 1). The first workshop concentrated on nuclear energy and explored its possible future scientific and technological developments, while the second workshop addressed renewable energies, energy systems and storage (Table 1). The papers presented in this Special Issue were written by experts who participated in the project and benefitted from the opportunities for international information sharing and discussion.

**Box 1:** EASAC steering committee for the Breakthrough study

Lennart Bengtsson, KVA; cochair, lennart.bengtsson@mpimet.mpg.de
Elisabeth Rachlew, KVA; cochair, erk@kth.se
Dick Hedberg, KVA; dickh@kva.se
Sven Kullander, KVA; (deceased January 2014)
Olle Inganäs, Linköping, Sweden; ois@ifm.liu.se
Villy Sundström, Lund, Sweden; villy.sundstrom@chemphys.lu.se
Eva-Mari Aro, Turku, Finland; evaaro@utu.fi
Ilkka Savolainen, Helsinki, Finland; ilkka.savolainen@vtt.fi (left June 2013)
Matthias Beller, Leibniz, Germany; Matthias.beller@catalysis.de
Thomas Hamacher, München, Germany; thomas.hamacher@tum.de
Johan Carlsson, JRC, The Netherlands; johan.carlsson@ec.europa.eu
Samuele Furfari, Brussels, Belgium; sfurfari@ulb.ac.be
Krzysztof Zmijewski, Warsaw, Poland; Krzysztof.zmijewski@interia.pl
Vicente Carabias, Switzerland; cahu@zhaw.ch
John Holmes, EASAC, United Kingdom; jholmes2@btinternet.com
Don MacElroy, Dublin, Ireland; don.macelroy@ucd.i.e.
Akos Horvath, Budapest, Hungary; horvath.akos@energia.mta.hu
Constantino Vayenas, Patras, Greece; cgvayenas@upatras.gr

**Table 1:** The project has included the following meetings besides the four meetings of the steering committee: *Workshop on the future of nuclear energy,* Greifswald, April 8–9, 2013 (http://www.easac.eu/energy/wg-low-carbon-energy.html) and *Workshop on renewables, storage and systems*, KVA, Stockholm, September 20–21, 2013 (http://www.kva.se/en/Science-in-Society/Energy-Committee/Breakthroughs-in-Sustainable-Energy/)

Researcher	Institution	Title of presentation
*Workshop on the future of nuclear energy*		
Hamid Aït Abderrahim	MOL, Belgium	Future Advanced Nuclear Systems And Role of MYRRHA
Hardo Bruhns	Düsseldorf, Germany	Framework aspects for the use of nuclear power in the longer-term future
Ákos Horváth	Budapest, Hungary	New projects in Eastern Europe and the sustainability of nuclear energy
Boris Kuteev	Moscow, Russia	Possible outcome of fusion-fission power plant by 2050 and beyond
Alex C. Mueller	CNRS, Paris, France	Pyroprocessing and fast reactors by 2050—reflections on pros and cons
Friedrich Wagner	IPP, Greifswald, Germany	More effective energy distribution on a European scale
Robert Wolf	IPP, Greifswald, Germany	Fusion research and Wendelstein 7-X
Friedrich Wagner	IPP, Greifswald, Germany	Options of nuclear fusion beyond 2050
*Workshop on renewables, storage and systems*		
Paul Alivisatos	Lawrence Berkeley National Laboratory, USA	Nanoscience and the future of the Global Carbon Cycle
Karl Leo	Technical University Dresden, Germany	Recent progress in organic solar cells: From a lab curiosity to a serious photovoltaic technology
Markus Antonietti	Max Planck Institute of Colloids and Interfaces, Germany	Lactid acid, ionic liquids and energy storage materials—Perspectives of Hydrothermal Biomass Upgrade
Eli Yablonovitch	University of California Berkeley, USA	Photovoltaics, high efficiency together with low cost
René J. Janssen	Technical University Eindhoven, The Netherlands	Efficient polymer solar cells and first steps beyond that
Frank Dimroth	Fraunhofer-Gesellschaft, Germany	Photovoltaic research for the support of European energy transition
Magnus Borgström	Lund University, Sweden	Nanowires with promise for high efficiency photovoltaics
Anders Hagfeldt	Uppsala University, Sweden	Hybrid inorganic–organic photovoltaics—HI-OPV
Klaas Hellingwerf	University of Amsterdam, The Netherlands	Cyanobacteria as the ultimate photo-catalysts of the conversion of carbon dioxide into chemical commodities and liquid fuel, driven by either sunlight or electricity
Per Gardeström	Umeå Plant Science Center, Sweden	Energy and green chemicals from forest products
Sascha Rexroth	Ruhr University Bochum, Germany	Rational design of cyanobacteria for hydrogen production
Vincent Artero	CEA, France	Molecular science for artificial photosynthesis: from bio-inspired catalysts to nanomaterials
Erwin Reisner	University of Cambridge, UK	Artificial photosynthesis with enzymes and synthetic catalysts integrated in nanostructured hybrid materials
Daniel Nocera	Harvard University, USA	The artificial leaf (was hindered to participate)
Styrbjörn Styring	Uppsala University, Sweden	Artificial photosynthesis
Michel Armand	The National Center for Scientific Research, France	Electrochemical energy storage, activity on all fronts
Thomas Hamacher	Technical University Munich, Germany	Integration of renewable energies: competition between storage, the power grid and flexible demand
Hermann-Josef Wagner	Ruhr University Bochum, Germany	Wind energy systems- present status and ecobalances
Godfrey Boyle	The Open University, UK	Renewables-intensive Energy Systems for the United Kingdom
Ujjval Vyas	Alberti Group, USA	The importance of failure and the future of renewable energy
Sture Larsson	Former Technical Director and deputy Director General at Svenska Kraftnät, the Swedish Power System Operator (TSO), Sweden	Requirements for system adaptions to intermittent energies

The main sources of energy supply addressed during the project were carbon-based fossil fuels, solar photovoltaics, biofuels and nuclear. Whilst energy efficiency was an essential issue throughout the discussions and special consideration was given to the energy efficiency of engines and appliances, particular attention was given to the future of electricity grids, electricity storage and fuel cells. Lastly, concerning energy consumption, there was an important focus on energy for transport.

One important conclusion from this project is that the energy issue should not be split up into independent contributions: electricity, heat, mechanical work, etc. The transformation to a largely CO_2_-free energy supply requires that the chemical energy forms are replaced predominantly by electricity. Even more than in the past, an energy policy and development strategy requires keeping in mind the total picture—energy generation, energy transportation and energy usage and each area calls for increased research. Even if a timespan for this transition of more than thirty years does seem long, we nevertheless have to conclude that fossil energy will still be in the energy mix for a long time globally. Therefore, we have to accept the unavoidable need to develop carbon capture and storage techniques, even if Europe could escape to employ this technology. MacElroy (2016) points out clearly the present situation and what research is needed for the future for closing the carbon cycle. Furthermore, the technological development in nuclear energy could alleviate the question of long-term storage of high level nuclear waste. Nuclear fusion research has the chance within the next decade to demonstrate the feasibility of this concept and to demonstrate that a fusion reactor could be an option in the long-term energy mix which is highlighted in the article by Horvath and Rachlew (2016).

Wind and solar power have shown a remarkable growth in many countries inside and outside Europe. In countries like Germany, the added installed power level matches peak demand. The efficiency of the solar cells has reached levels where solar cell panels could give considerable contributions to the energy mix in most European countries. Still, new materials might emerge with even better photovoltaic properties. Several basic science research areas within the fields of solar and biofuels are highlighted. The article by Inganäs and Sundström (2016) highlights the possible development for photovoltaics to enter in a large scale with more efficient, resilient and economic solar panels and takes a look into the research development of the materials needed. The scene of the many functionalities of biofuels is painted by Aro (2016) in her article, which highlights where worldwide research is flourishing.

The introduction of intermittent electricity sources into the production requires more planning and changes to the distribution net which is modelled and discussed in the paper by Kuhn et al. (2016). In many countries most of the fossile contributions come from the transport sector which would need a transformation to electric vehicles and/or a combination with fuel cells. Both these issues are discussed in the articles by Furfari (2016) and by Niakolas et al. (2016).

Some basic science and major technology research areas have not been included, such as development of chemical and electrical storage systems, and development of new materials (for nuclear reactors, for batteries, for solar panels, for cables), in order to focus this issue more towards the generation of the energy needed for the future.

In summary, the seven papers included give an overview of fields in energy research which could promise essential progress in low-carbon energy supply and use.
